# Growth and weight gain in children with juvenile idiopathic arthritis: results from the ReACCh-Out cohort

**DOI:** 10.1186/s12969-017-0196-7

**Published:** 2017-08-22

**Authors:** Jaime Guzman, Tristan Kerr, Leanne M. Ward, Jinhui Ma, Kiem Oen, Alan M. Rosenberg, Brian M. Feldman, Gilles Boire, Kristin Houghton, Paul Dancey, Rosie Scuccimarri, Alessandra Bruns, Adam M. Huber, Karen Watanabe Duffy, Natalie J. Shiff, Roberta A. Berard, Deborah M. Levy, Elizabeth Stringer, Kimberly Morishita, Nicole Johnson, David A. Cabral, Maggie Larché, Ross E. Petty, Ronald M. Laxer, Earl Silverman, Paivi Miettunen, Anne-Laure Chetaille, Elie Haddad, Lynn Spiegel, Stuart E. Turvey, Heinrike Schmeling, Bianca Lang, Janet Ellsworth, Suzanne E. Ramsey, Johannes Roth, Sarah Campillo, Susanne Benseler, Gaëlle Chédeville, Rayfel Schneider, Shirley M. L. Tse, Roxana Bolaria, Katherine Gross, Debbie Feldman, Bonnie Cameron, Roman Jurencak, Jean Dorval, Claire LeBlanc, Claire St. Cyr, Michele Gibbon, Rae S. M. Yeung, Ciarán M. Duffy, Lori B. Tucker

**Affiliations:** 1 From British Columbia Children’s Hospital and University of British Columbia, Vancouver, Canada; 2 Department of Pediatrics University of British Columbia, Vancouver, Canada; 3 Stollery Children’s Hospital and University of Alberta, Edmonton, Canada; 4 Alberta Children’s Hospital and University of Calgary, Calgary, Canada; 5 Royal University Hospital and University of Saskatchewan, Saskatoon, Canada; 6 Department of Pediatrics and Child Health, University of Manitoba, Winnipeg, Canada; 7 London Health Sciences Centre and Western University, London, Canada; 8 McMaster University, Hamilton, Canada; 9 Hospital for Sick Children and University of Toronto, Toronto, Canada; 10 Children’s Hospital of Eastern Ontario and University of Ottawa, Ottawa, Canada; 11 McGill University Health Centre and McGill University, Montreal, Canada; 12 Centre Hospitalier Universitaire Ste. Justine and Université de Montréal, Montréal, Canada; 13 Université de Montréal, Montréal, Canada; 14 Centre Hospitalier Universitaire de Sherbrooke and Université de Sherbrooke, Sherbrooke, Canada; 15 Centre Hospitalier Universitaire de Laval and Université Laval, Quebec, Canada; 16 IWK Health Centre and Dalhousie University, Halifax, Canada; 17 Janeway Children’s Health and Rehabilitation Centre and Memorial University, Saint John, ’s Canada; 18 Shands Children’s Hospital and University of Florida, Gainesville, USA; 19 Division of Pediatric Rheumatology, BC Children’s Hospital, 4500 Oak St, Suite K4-122, Vancouver, BC V6H 3N1 Canada

**Keywords:** Juvenile arthritis, Growth, Obesity, Corticosteroids

## Abstract

**Background:**

With modern treatments, the effect of juvenile idiopathic arthritis (JIA) on growth may be less than previously reported. Our objective was to describe height, weight and body mass index (BMI) development in a contemporary JIA inception cohort.

**Methods:**

Canadian children newly-diagnosed with JIA 2005–2010 had weight and height measurements every 6 months for 2 years, then yearly up to 5 years. These measurements were used to calculate mean age- and sex-standardized Z-scores, and estimate prevalence and cumulative incidence of growth impairments, and the impact of disease activity and corticosteroids on growth.

**Results:**

One thousand one hundred forty seven children were followed for median 35.5 months. Mean Z-scores, and the point prevalence of short stature (height < 2.5th percentile, 2.5% to 3.4%) and obesity (BMI > 95th percentile, 15.8% to 16.4%) remained unchanged in the whole cohort. Thirty-three children (2.9%) developed new-onset short stature, while 27 (2.4%) developed tall stature (>97.5th percentile). Children with systemic arthritis (*n* = 77) had an estimated 3-year cumulative incidence of 9.3% (95%CI: 4.3–19.7) for new-onset short stature and 34.4% (23–49.4) for obesity. Most children (81.7%) received no systemic corticosteroids, but 1 mg/Kg/day prednisone-equivalent maintained for 6 months corresponded to a drop of 0.64 height Z-scores (0.56–0.82) and an increase of 0.74 BMI Z-scores (0.56–0.92). An increase of 1 in the 10-cm physician global assessment of disease activity maintained for 6 months corresponded to a drop of 0.01 height Z-scores (0–0.02).

**Conclusions:**

Most children in this modern JIA cohort grew and gained weight as children in the general population. About 1 in 10 children who had systemic arthritis, uncontrolled disease and/or prolonged corticosteroid use, had increased risk of growth impairment.

**Electronic supplementary material:**

The online version of this article (doi:10.1186/s12969-017-0196-7) contains supplementary material, which is available to authorized users.

## Background

Juvenile idiopathic arthritis (JIA) is the most common rheumatic disease of childhood [1], and the seven JIA categories have different characteristics and outcomes [2, 3]. Chronic inflammation in children with JIA may lead to growth delay and poor weight gain, while use of corticosteroids to control inflammation may lead to growth delay and excessive weight gain. Our knowledge about growth in children with JIA is largely based on older cross-sectional studies and retrospective cohorts from single centres [4, 5]. These studies included 20 to 200 children each and reported frequent growth impairments (a deviation from healthy growth standards in height, weight or body mass index, BMI) [5]. More recently, synthetic disease-modifying antirheumatic drugs (DMARDS) and biologic medications, which help control inflammation and reduce the need for corticosteroids, have been reported to mitigate growth impairment [6–9]. Yet, as recently as 2011, significant height growth delay was reported in oligoarthritis, a usually mild JIA category rarely treated with systemic corticosteroids [10].

In this study, we used data from a prospective multicentre inception cohort of over one thousand Canadian children to determine 1) height, weight and BMI trajectories over time in children with JIA managed with modern treatments; 2) their risk of growth impairments; and 3) the impact of disease activity and corticosteroid use on growth.

## Methods

### Subjects

Data from the Research in Arthritis in Canadian Children emphasizing Outcomes (ReACCh-Out) cohort were used. ReACCh-Out was designed to document disease outcomes in usual practice [3, 11]. It enrolled 1497 patients newly diagnosed with JIA between 2005 and 2010 at all 16 Canadian pediatric rheumatology centres. JIA category was assigned using accepted criteria within 6 months of enrolment [2, 3]. Study visits were scheduled at enrolment and at 6, 12, 18, 24, 36, 48, and 60 months thereafter. The study was approved by research ethics boards at each participating institution. Parents provided informed written consent, and patients provided assent where appropriate. This report includes children recruited within 6 months of diagnosis who had at least two available height and weight records as of May 30th, 2012.

### Measures of growth and weight gain

Standing height and weight were measured at each study visit as per usual clinic practices. We calculated age- and sex-standardized scores (Z-scores, 0 = mean of healthy children, 1 = one standard deviation in healthy children) [12], for height, weight, and BMI for each child using standards for healthy growth endorsed by the Canadian Pediatric Endocrinology Group [13]. These are equivalent to the 2007 World Health Organization standards, except that standards for both weight and BMI are provided up to age 18 years [14].

Growth impairments were defined as follows:

*Short stature:* a height below the 2.5th percentile for age and sex (corresponding to a Z-score < −2.0) [15].

*Tall stature:* a height above the 97.5th percentile for age and sex.

*Obesity:* a BMI above the 95th percentile for age and sex [12].

*Thinness:* a BMI below the 5th percentile for age and sex.

*Growth delay:* a decrease of 1.0 or more in height Z-score at a given visit, relative to the Z-score of that child at enrolment.

*Growth acceleration:* an increase of 1.0 or more in height Z-score at a given visit, relative to the Z-score of that child at enrolment.

*Excessive weight gain:* an increase of 1.0 or more in BMI Z-score at a given visit, relative to the Z-score of that child at enrolment.

*Poor weight gain:* a decrease of 1.0 or more in BMI Z-score at a given visit, relative to the Z-score of that child at enrolment.

These definitions and cut-offs have been used in previous studies, but they are not universally accepted. The Z-score at enrolment was calculated using the first available measurements of height and weight (a median of 0.5 months after diagnosis), representing our best estimate of the child’s baseline growth. A change of 1.0 Z-scores from enrolment was chosen because it roughly corresponds to the crossing of a percentile channel on growth charts [16], and it had been used in a previous study [10].

### Other measures

Disease activity and medication use were recorded at each study visit, and at clinic visits in-between study visits. Disease activity was reported by the pediatric rheumatologist using a standard 10-cm horizontal visual analogue scale from 0 = inactive disease to 10 = very active disease [17]. Early treatment responders (versus non-early responders) were defined as those who attained clinically inactive disease within a year of diagnosis [3, 17].

The name and current dose of oral and intravenous corticosteroids recorded at each clinic visit were used to calculate mg/kg/day in prednisone equivalent using published conversion factors [18, 19], and the most recently recorded patient’s weight. Children were categorized as non-users of systemic corticosteroids, transitory users (3 months or less) or prolonged users (>3 months of cumulative use during the study), irrespective of the doses or frequency of administration; intraarticular corticosteroid administration was not considered in this categorization.

The education level of the parent with the highest level of education was used as a proxy for socio-economic status and self-reported ethnicity was recorded using Statistics Canada standard categories.

### Statistical analysis

Analyses were conducted using STATA 12 software [StataCorp LP, College Station, Texas]. Trajectories of the mean Z-score over time were charted using locally weighted scatter plot smoothing with a band width of 0.6 to produce lines reflecting observed data without imposing a particular shape. Kaplan Meier survival methods were used to estimate the cumulative incidence of new-onset growth impairments. These consider only the first occurrence of growth impairment, and do not reflect persistence or resolution of the impairment. Because the number of subjects in some JIA categories was small beyond three years, we show plots only to three years after diagnosis. The log-rank test was used to compare survival curves. Mixed effects models assessed the impact of cumulative disease activity and corticosteroid use in the previous six months on Z-scores, after adjusting for parental education, ethnicity and JIA category. Models were implemented in STATA as growth curve models [20] with random intercept, random slope and a quadratic term for time since diagnosis (see Additional file 1 for details).

Sensitivity analyses to assess the robustness of our findings included use of alternative growth charts and measures of disease activity [21], analyses in children of pre-pubertal age (defined as <7 years at diagnosis) and calculation of cumulative incidence of reciprocal “growth impairments” (e.g. tall stature and growth acceleration) to assess to what extent our results were due to normal variability and the choice of cut-off, rather than true impairments.

## Results

**Fig. 1:**
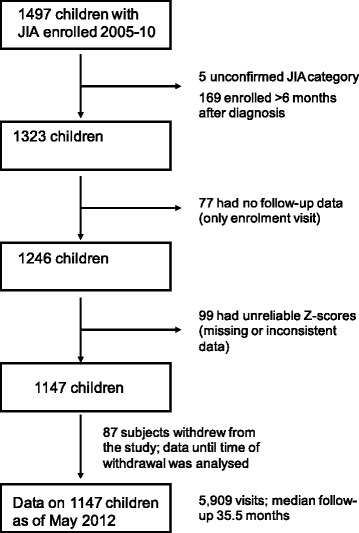
Subject eligibility and disposition

A total of 1147 children were included (Table 1). The reasons for ineligibility and subject disposition are shown in Fig. 1. The median age at diagnosis was 9.5 years (25th, 75th centile: 4, 13) and 64% were female. The median time from diagnosis to enrolment was 0.5 months (0, 1.9). Height and weight measurements were available on 5909 visits for a median follow-up of 35.5 months (25th, 75th centile: 23, 49). Additional file 1: Table S1 shows subjects with data at each visit.

**Table 1:** Characteristics of patients by JIA category^a^

Characteristic	All (*N* = 1147)	Systemic Arthritis (*n* = 77)	Oligoarthritis (*n* = 443)	RF-negative polyarthritis (*n* = 232)	RF-positive polyarthritis (*n* = 44)	Psoriatic arthritis (*n* = 70)	Enthesitis-related Arthritis (*n* = 164)	Undifferentiated (*n* = 117)
% Female	64.0	47.4	70.2	77.6	95.5	67.1	25.0	68.4
Age at diagnosis (yrs)	9.5 (4.0, 13.1)	6.6 (3.1, 12.2)	6.4 (3.0, 11.5)	9.1 (3.4, 12.7)	12.8 (9.4, 15.2)	11.6 (5.3, 13.6)	13.1 (10.8,14.5)	9.5 (3.7, 13.1)
Disease duration (months)	5.8 (3.1, 10.9)	2.9 (1.9, 5.4)	5.3 (3.0, 8.9)	6.1 (3.4, 12.4)	5.5 (3.6, 10.6)	6.5 (3.0, 13.6)	8.9 (4.2,17.3)	5.9 (3.5, 9.0)
Follow-up (months)	35.5 (23,49)	40.7 (24,52)	35.0 (23,49)	36.7 (24,52)	33.1 (23, 49)	34.5 (18, 49)	30.9 (19, 40)	35.7 (23, 48)
Height Z-score at enrolment	0.02 (−0.70, 0.82)	0.09 (−0.69, 0.91)	0.06 (−0.65, 0.83)	−0.13 (−0.77, 0.58)	0.02 (−0.59, 0.69)	0.12 (−0.68, 0.99)	0.16 (−0.52, 0.98)	−0.19 (−0.92, 0.62)
Weight Z-score at enrolment	0.31 (−0.37, 1.06)	0.58 (0.04, 1.25)	0.25 (−0.35, 0.90)	0.17 (−0.53, 0.94)	0.16 (−0.83, 0.94)	0.66 (−0.26, 1.4)	0.61 (−0.52, 1.4)	0.29 (−0.53, 0.96)
BMI percentile at enrolment	66 (34, 89)	80.4 (62, 90)	62.6 (35, 87)	64.6 (34, 87)	55.8 (18, 88)	75.3 (46, 96)	65.8 (28, 93)	67.0 (38, 88)
Disease activity (mm)	28 (12, 50)	37 (13, 62)	19 (8, 34)	46 (27, 66)	52 (26, 71)	23 (11, 51)	30 (12, 46)	26 (12, 48)
ESR, mm/h^b^	18 (7, 36)	58 (25, 89)	17 (7, 31)	18 (8, 36)	36 (16, 53)	15 (8, 36)	10 (4, 24)	16 (5, 35)
*Treatments* ^c^								
DMARDs (%)	55.7	71.1	33.9	84.9	97.7	50.0	58.7	53.8
Biologics (%)	11.7	34.2	3.6	15.9	40.9	7.1	12.2	10.3
Corticosteroid joint injections (%)	44.3	17.1	55.5	43.5	43.2	41.4	31.4	41.9
Systemic corticosteroids (%)	18.2	85.5	9.3	28.0	63.6	17.1	23.8	23.1
Corticosteroids >3 months (%)	10.6	65.0	0.9	10.8	29.5	2.9	9.3	11.1
*Ethnicity* ^d^								
British (%)	50.2	41.9	44.7	54.1	44.2	60.6	54.6	53.5
French (%)	31.2	22.1	35.8	26.1	18.6	34.8	22.7	42.1
Indigenous Canadian (%)	8.1	5.8	8.9	7.7	16.3	6.1	5.5	8.8
Other (%)	25.4	26.7	21.8	31.5	34.9	16.7	30.7	16.7
*Parental education* ^e^								
Less than high school (%)	2.01	1.6	1.9	2.9	0	1.6	2.6	1.0
High school/some postsecondary (%)	50.24	53.1	45.8	51.7	61.9	54.1	51.6	56.4
University/postgraduate degree (%)	47.75	45.3	51.6	46.4	38.1	44.3	45.8	42.6
*Inactive disease within 1 year of diagnosis (%)*	49.5	44.7	65.48	36.6	22.7	42.9	37.8	39.3

^a^Numbers are median and (25th, 75th centiles) unless otherwise specified

^b^ESR was obtained in 88% of the cohort at enrolment

^c^Treatments received at any time during the study

^d^Self-reported ethnicity was available in 96% of the cohort. Other ethnicity includes Asian, African, Latin American and other European ethnicities. The sum of ethnicities may add to more than 100% because each child could report up to 6 ethnic backgrounds

^e^The level of education of the parent who had a higher level of education. Parental education for at least one parent was obtained in 93% of the cohort at enrolment

### Height, weight and BMI trajectories

**Fig. 2:**
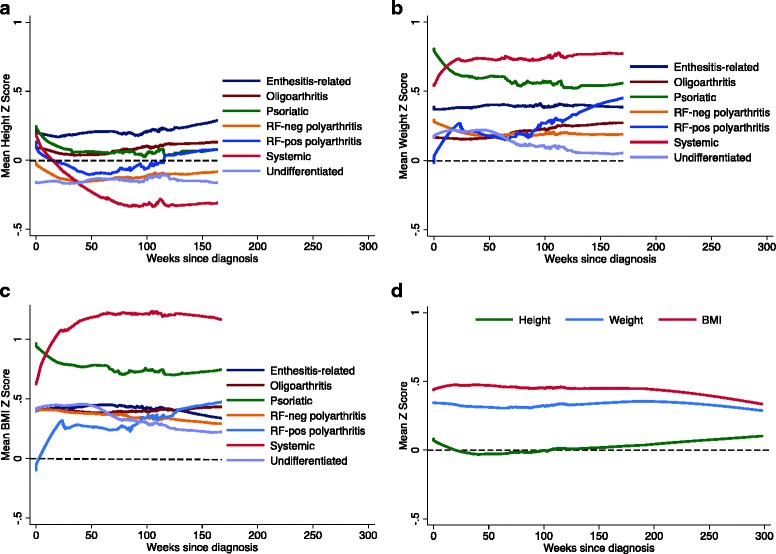
Smoothed trajectories of mean Z-scores. Shown are trajectories of (**a**) height, (**b**) weight, and (**c**) BMI for each JIA category in the 3 years after diagnosis. Panel (**d**) shows Z-score trajectories for the whole cohort. Trajectories of the mean Z-score were charted using locally weighted scatter plot smoothing with a band width of 0.6. The black dashed horizontal line represents healthy growth standards

Figure 2 shows trajectories of height, weight, and BMI mean Z-scores for the whole cohort and for each JIA category. Mean height Z-scores remained unchanged for the whole cohort (Fig. 2d). Children with enthesitis-related arthritis had an estimated mean height Z-score at diagnosis of +0.2 and remained unchanged over 3 years. In contrast, children with systemic arthritis also had an estimated mean height Z-score at diagnosis of +0.2 but it decreased and did not return to baseline within 3 years. Using quadratic fit equations the corresponding 95% CI become non-overlapping by one year (Additional file 1: Fig. S1).

Mean weight and BMI Z-scores in the whole cohort and in the most frequent JIA categories (oligoarthritis, RF-negative polyarthritis, enthesitis-related, undifferentiated) remained stable over time (Fig. 2). These parameters increased during the first year after diagnosis in children with systemic JIA and RF-positive polyarthritis, but their estimates at diagnosis were different: a mean BMI Z-score of +0.6 for systemic and −0.1 for RF-positive polyarthritis. Mean values of weight and BMI Z-scores of children with psoriatic arthritis were high at enrolment and decreased over time.

### Growth impairments

**Fig. 3:**
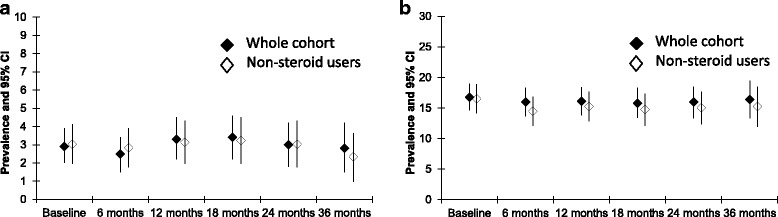
Point prevalence of short stature and obesity in the whole cohort. Shown are the point prevalences of **a**) short stature and **b**) obesity in the whole cohort at every study visits for the 3 years after enrolment, and after excluding children who received corticosteroids. The vertical lines represent 95%CI

The point prevalence of short stature in the whole cohort remained stable during the study (2.5 to 3.4%, Fig. 3). Thirty-three children (2.9%) had new-onset short stature (<2.5th percentile), while 27 (2.4%) had new-onset tall stature (>97.5th percentile) during the study. Ninety-three children (8.1%) had growth delay, while 64 (5.6%) had growth acceleration. The estimated cumulative incidence of growth delay within 3 years of diagnosis for the whole cohort was 8.5% (95%CI: 6.8–10.6, Table 2). It varied from 5.7% to 9.9% across JIA categories, except for systemic arthritis (22.5%, 14.2–34.7).

**Table 2:** Estimates of the cumulative incidence (%) of growth delay, short stature, excessive weight gain and obesity within 1 and 3 years after diagnosis in seven JIA categories

JIA Category	Growth delay		Short stature		Excessive weight gain		Obesity	
	1-year cumulative incidence (95% CI)	3-year cumulative incidence (95% CI)	1-year cumulative incidence (95% CI)	3-year cumulative incidence (95% CI)	1-year cumulative incidence (95% CI)	3-year cumulative incidence (95% CI)	1-year cumulative Incidence (95% CI)	3-year cumulative Incidence (95% CI)
All (*N* = 1147)	3.2 (2.4, 4.5)	8.5 (6.8, 10.6)	1.2 (0.7, 2.1)	3.0 (2.1, 4.4)	4.8 (3.7, 6.2)	10.7 (8.8, 12.9)	5.2 (4.0, 6.9)	10.8 (8.8, 13.2)
Systemic arthritis (*n* = 77)	6.8 (2.9, 15.7)	22.5 (14.2, 34.7)	1.4 (0.2, 9.8)	9.3 (4.3, 19.7)	17.2 (10.4, 27.8)	28.1 (19.0, 40.3)	22.7 (13.9, 35.9)	34.4 (23.0, 49.4)
Oligoarthritis (*n* = 443)	3.8 (2.3, 6.1)	7.8 (5.3, 11.3)	1.2 (0.5, 2.9)	2.3 (1.2, 4.4)	3.8 (2.3, 6.1)	10.5 (7.6, 14.4)	3.3 (1.9, 5.7)	8.2 (5.5, 12.1)
RF-negative polyarthritis (*n* = 232)	4.0 (2.1, 7.6)	7.4 (4.5, 12.0)	0.9 (0.2, 3.6)	0.9 (0.2, 3.6)	3.6 (1.8, 7.0)	8.9 (5.6, 14.1)	3.5 (1.7, 7.2)	8.5 (5.2, 13.8)
RF-positive polyarthritis (*n* = 44)	---	9.9 (3.1, 28.7)	---	4.3 (0.6, 27.1)	9.1 (3.5, 22.6)	15.0 (7.2, 31.9)	---	5.9 (0.8, 35.0)
Psoriatic arthritis (*n* = 70)	2.9 (0.7, 10.9)	9.5 (4.0, 21.8)	2.9 (0.7, 11.1)	9.5 (3.9, 22.0)	3.0 (0.7, 11.4)	5.0 (1.6, 14.9)	4.1 (1.0, 15.5)	11.7 (5.0, 26.0)
Enthesitis-related arthritis (*n* = 164)	1.2 (0.3, 4.7)	7.2 (3.6, 14.4)	0.6 (0.1, 4.5)	1.6 (0.4, 6.3)	3.7 (1.7, 8.1)	8.3 (4.5, 14.9)	5.4 (2.6, 10.9)	9.7 (5.4, 17.2)
Undifferentiated (*n* = 117)	1.9 (0.5, 7.2)	5.7 (2.4, 13.5)	1.9 (0.5, 7.4)	3.6 (1.1, 11.4)	3.6 (1.4, 9.3)	7.6 (3.9, 14.7)	8.4 (4.3, 16.0)	14.1 (8.4, 23.1)

Growth delay: a decrease of 1.0 or more in height Z-score relative to the z-score of that child at enrolment. Short stature: a height below the 2.5th percentile for age and sex. Excessive weight gain: an increase of 1.0 or more in BMI Z-score relative to the z-score of that child at enrolment. Obesity: a BMI above the 95th percentile for age and sex

--- = Cumulative incidence could not be calculated as no child had experienced the growth impairment at this time

The point prevalence of obesity in the whole cohort remained stable during the study (15.8 to 16.8%, Fig. 3). Ninety-seven children (8.5%) developed new onset obesity, while 43 (3.7%) developed new onset thinness. The estimated cumulative incidence of obesity within three years of diagnosis was 10.8% (95%CI: 8.8–13.2, Table 2); it varied from 5.9% to 14.1% across JIA categories, except for systemic arthritis (34.4%, 23.0–49.4). The estimated cumulative incidence of excessive weight gain within 3 years of diagnosis varied from 5% to 15% across JIA categories, except for systemic arthritis (28.1%, 19.0–40.3).

### Impact of corticosteroids and disease activity

In adjusted mixed effects models, a dose of 1 mg/kg/day prednisone equivalents maintained for 6 months corresponded to a decrease of 0.64 in height Z-scores (95%CI: 0.56–0.82) and an increase of 0.74 in BMI Z-scores (0.56–0.92) (Table 3). Additional file 1: Fig. S2 shows trajectories of mean corticosteroid dose for each JIA category. Most children received no systemic corticosteroids during the study (*n* = 943, 81.7%), while 88 (7.6%) were transitory users and 123 (10.6%) were prolonged users (>3 months). The estimated cumulative incidence of short stature among prolonged corticosteroid users was 6.6% within 3 years, compared to 2.6% for non-users (*p* = 0.07, log-rank test for survival curves). The estimated cumulative incidence of obesity among prolonged corticosteroid users was 25% within 3 years, compared to 8.5% for non-users (*p* < 0.0001, log-rank test for survival curves, Additional file 1: Fig. S3).

**Table 3:** Impact of corticosteroid use, disease activity and covariates on height Z-scores and BMI Z-scores

	*Impact on height Z-scores*				*Impact on BMI Z-scores*			
Variable	Unadjusted^a^ (95% CI)	*p* value	Adjusted (95% CI)	*p* value	Unadjusted (95% CI)	*p* value	Adjusted (95% CI)	*p* value
Cumulative corticosteroids^b^	−0.69 (−0.82, −0.56)	<0.01	−0.64 (−0.77, −0.50)	<0.01	0.79 (0.63, 0.96)	<0.01	0.74 (0.56, 0.92)	<0.01
Cumulative disease activity^c^	−0.02 (−0.03, −0.01)	<0.01	−0.01 (−0.02, 0.00)	0.04	0.00 (−0.01, 0.01)	0.49	−0.01 (−0.02, 0.00)	0.17
Parental education^d^								
Less than high school	Reference		Reference		Reference		Reference	
High school/secondary	0.29 (−0.18, 0.77)	0.23	0.31 (−0.18, 0.80)	0.21	0.005 (−0.50, 0.51)	0.98	0.09 (−0.43, 0.61)	0.73
Some post-secondary	0.34 (−0.12, 0.80)	0.15	0.39 (−.08, 0.87)	0.11	−0.01 (−0.50, 0.48)	0.96	0.09 (−0.41, 0.60)	0.72
University degree	0.32 (−0.14, 0.78)	0.18	0.36 (−0.11, 0.84)	0.14	−0.17 (−0.66, 0.31)	0.49	−0.08 (−0.58, 0.43)	0.77
Postgraduate degree	0.40 (−0.08, 0.89)	0.10	0.43 (−0.07, 0.93)	0.09	−0.25 (−0.76, 0.26)	0.33	−0.11 (−0.64, 0.42)	0.68
Primary Ethnicity^e^								
British	Reference		Reference		Reference		Reference	
French	−0.09 (−0.26, 0.09)	0.32	−0.11 (−0.29, 0.07)	0.24	−0.36 (−0.55, −0.17)	<0.01	−0.33 (−0.52, −0.13)	<0.01
Indigenous Canadian	0.15 (−0.15, 0.45)	0.34	0.15 (−0.16, 0.46)	0.33	−0.05 (−0.37, 0.27)	0.76	−0.03 (−0.36, 0.30)	0.84
Other European	0.03 (−0.14, 0.19)	0.74	0.03 (−0.14, 0.20)	0.75	−0.31 (−0.49, −0.13)	<0.01	−0.23 (−0.41, −0.05)	0.01
South Asian	0.33 (0.0, 0.66)	0.05	0.34 (0.0, 0.67)	0.05	−0.13 (−0.48, 0.22)	0.47	−0.13 (−0.49, 0.23)	0.48
Other	−0.07 (−0.27, 0.13)	0.47	−0.03 (−0.24, 0.18)	0.75	−0.23 (−0.44, −0.02)	0.03	−0.13 (−0.35, 0.09)	0.25
JIA categories								
Oligoarthritis	Reference		Reference		Reference		Reference	
RF-neg polyarthritis	−0.17 (−0.34, −0.01)	0.04	−0.14 (−0.32, 0.03)	0.10	−0.01 (−0.18, 0.17)	0.96	0.00 (−0.18, 0.19)	0.99
Enthesitis-related	0.17 (−0.01, 0.36)	0.07	0.21 (0.01, 0.40)	0.04	0.08 (−0.12, 0.27)	0.45	−0.04 (−0.25, 0.18)	0.74
Systemic	−0.36 (−0.61, −0.10)	0.01	−0.23 (−0.51, 0.05)	0.10	0.68 (0.41, 0.95)	<0.01	0.44 (0.15, 0.74)	<0.01
Psoriatic	0.04 (−0.23, 0.30)	0.79	0.16 (−0.12, 0.44)	0.26	0.41 (0.13, 0.69)	<0.01	0.31 (0.01, 0.61)	0.05
RF-pos polyarthritis	−0.13 (−0.46, 0.19)	0.43	−0.03 (−0.36, 0.31)	0.88	−0.13 (−0.47, 0.21)	0.46	−0.25 (−0.60, 0.10)	0.17
Undifferentiated	−0.18 (−0.39, 0.04)	0.11	−0.11 (−0.33, 0.12)	0.37	0.03 (−0.20, 0.25)	0.82	0.02 (−0.22, 0.26)	0.86

^a^Unadjusted Beta coefficients and (95% CI) were obtained from mixed effect models including only one independent variable at a time. Adjusted beta coefficients are from a mixed effects model where cumulative corticosteroids in previous 6 months, cumulative disease activity in previous 6 months, parental education, ethnicity and JIA category were entered together. Cumulative prednisone and disease activity were time-variant variables. All mixed effects models were run by subject and included random intercept, random slope and a quadratic term for time since diagnosis in weeks. They can be represented by: dependent variable = b1*constant + b2*time + b3*time square + b4* first independent variable + b5* second independent variable

^b^Cumulative corticosteroids were calculated as area under the curve of daily prednisone equivalents per kilogram of body weight recorded at each clinic visit, using the trapezoid method; a one unit change corresponds to an increase of 1 mg/Kg of prednisone equivalents per day sustained for 6 months. For the first study visit, corticosteroids were assumed to start at diagnosis

^c^Cumulative disease activity was calculated as area under the curve of the physician global assessment of disease activity recorded at each clinic visit, using the trapezoid method; a one unit change corresponds to an increase of 1 cm in the physician global assessment sustained for 6 months. For the first study visit the physician global assessment was assumed to be zero the day before disease onset

^d^The level of education of the parent who had a higher level of education

^e^The ethnic group listed first; up to six ethnic groups could be listed for each child as defined by Statistics Canada

In adjusted models, a 1 cm increase in the 10 cm physician global assessment of disease activity maintained for 6 months was associated with a decrease of 0.01 in height Z-scores (95%CI: 0.00–0.02) (Table 3). The association between disease activity and changes in BMI Z-scores was not significant. Mean disease activity decreased in all JIA categories after diagnosis (Additional file 1: Fig. S2) and 567 children (49.5%) attained inactive disease within the first year. Early treatment responders and non-early treatment responders had similar probability of growth impairments (Additional file 1: Fig. S4).

### Sensitivity analyses

Using the Centers for Disease Control growth charts had minimal impact on our estimates of growth impairments, except for a lower cumulative incidence of obesity within 3 years of diagnosis of 7.5% (95%CI: 5.9–9.6) (Additional file 1: Table S2). Calculations for reciprocal “growth impairments” (growth acceleration, tall stature, poor weight gain and thinness) are shown in Additional file 1: Table S3 and suggest a substantial portion of the incidence of growth impairments reported in Table 2 is due to normal variability and our choice of cut-offs rather than to the disease; for example, the 3-year cumulative incidence of tall stature for the whole cohort was 2.3%, compared to 3.0% for short stature. Children younger than 7 years at diagnosis had higher cumulative incidence of growth impairments, relative to the whole cohort, and a different distribution of JIA categories (Additional file 1: Table S4). Use of the active joint count or the Juvenile Arthritis Disease Activity Score [21], as alternative measures of disease activity, did not substantially change our findings (Additional file 1: Table S5).

## Discussion

We used data from a large prospective inception cohort of Canadian children with JIA to estimate impact of the disease and its treatment on height, weight and BMI development. For children in the most frequent JIA categories (oligoarthritis, RF-negative polyarthritis, enthesitis-related arthritis and undifferentiated arthritis, 83.5% of the cohort) the impact was negligible. On the other hand, children with systemic arthritis showed a decrease in height Z-scores and increase in BMI Z-scores, with no apparent return to baseline within 3 years. To put things in perspective, the decrease in mean height Z-scores in children with systemic JIA corresponds to approximately 2 cm for a 9 year old child. Children with RF-positive polyarthritis or psoriatic arthritis had relatively minor, but measurable, risk of growth impairments. For the whole cohort, point prevalence for short stature and obesity remained unchanged over the 3 years after diagnosis, suggesting the observed growth impairments affected too few children and/or were too short-lived to affect the overall prevalence.

Most children received no systemic corticosteroids, in those who did, larger doses of corticosteroids were clearly associated with lower height and higher BMI Z-scores. A similar impact of corticosteroids on BMI has been shown in children with a variety of rheumatic diseases [22]. In our cohort, most children with systemic JIA received high-dose corticosteroids, which likely mediated their observed growth impairments. Increased disease activity was associated with lower height Z-scores but the impact was minor, relative to that of corticosteroids.

Our original protocol considered a change of 1.0 Z-scores relative to baseline indicative of growth impairment because this roughly corresponds to crossing a percentile channel in growth charts [16], and it had been used in a previous study [10]. However, we later realized this definition may lead to apparent growth impairments even in healthy children. Growth in healthy children is complex and may have periods of relative stasis or rapid growth [23]. This may result in apparent delay or acceleration of growth when comparing a child to normative growth curves [12]. For example, children with enthesitis-related arthritis in our cohort had a virtually flat trajectory of mean height Z-score (i.e. they grew at the same pace than healthy children), but over 3 years they still had a 7.2% cumulative incidence of growth delay and a 7.7% cumulative incidence of growth acceleration. We believe cumulative incidences of this level using the 1.0 Z-score cut-off likely reflect normal periods of relative stasis or rapid growth, rather than true growth impairments. This could only be confirmed by following healthy children at the same time intervals and applying the 1.0 Z score cut-off; we are not aware of any such study.

Defining growth impairments as a change of 1.0 Z scores from baseline uses the child as his/her own control and allows identification of substantial changes in growth that may not be apparent when using the extreme limits (e.g. below 2.5th percentile or above 95th percentile). Nevertheless, some such changes may be desirable. For example, a child who is obese at baseline may drop more than 1.0 BMI Z-scores in follow-up and become closer to his/her ideal BMI (by our definition this is “poor weight gain”).

Although the World Health Organization has proposed standard terminology to describe growth impairments [15], it has not been universally adopted. The recommended term for a stature <−2.0 Z-scores is ‘stunting’, but we chose to use the term short stature instead, as we felt the implication that 2.5% of healthy children have stunted growth had overtly negative connotations. At the end, we chose to use neutral descriptive terms and most importantly, we defined them explicitly.

The high prevalence and cumulative incidence of obesity observed in our cohort may be a reflection of the current epidemic of obesity among North American children. An obesity point prevalence of 10.5 to 16.3% has been reported among Canadian children aged 5 to 11 years old [24], and new onset obesity has been reported in 11.9% of children in the United States between the ages of 5 and 14 years old [25]. However, children with systemic arthritis in our cohort had a three times higher incidence of obesity than these reported population values.

There were some interesting differences across JIA categories in growth parameters at enrolment (Table 1). These may reflect disease impact between symptom onset and enrolment, but they should be interpreted with caution as chance alone may explain these differences. One could postulate for example that the mean weight Z-score of 0.31 at enrolment for the whole cohort represents the current obesity epidemic among Canadian children, and that the mean z-score of 0.16 for RF-positive polyarthritis reflects weight loss during the nearly six months from symptom onset, while the mean Z-score of 0.58 for systemic JIA reflects corticosteroid-associated weight gain during the 2.9 months since symptom onset. Again, one ought to be careful not to over-interpret these differences given the large variability in growth parameters across children and small sample sizes in some JIA categories in this cohort. Of note, children with psoriatic arthritis had the highest mean weight Z-score of 0.66 at enrolment and they rarely, if ever, used corticosteroids.

Two published retrospective cohorts are of particular relevance to our study. In a study of 67 Canadian children with JIA, Liem and Rosenberg reported that relative to children with oligoarthritis, a lower height Z-score was apparent in the second year after diagnosis for children with systemic arthritis [26]. A similar change was apparent from the first year in our study. Children with RF-positive polyarthritis tended to have negative Z-scores, while children with RF-negative polyarthritis tended to have positive Z-scores [26]. In our study, children with RF-negative polyarthritis had practically normal growth while those with RF-positive polyarthritis had measurable, but mild growth impairments.

In the second study, Padeh et al. reported on 95 children with oligoarticular JIA in a single centre in Israel. After a mean follow-up of 6 years they reported “severe growth retardation” (a decrease in height Z-score of 1.0 or more relative to baseline) in 11.6% of children [10]. This estimate is compatible with our 3-year cumulative incidence of growth delay of 7.8% using the same cut-off, but as stated above, we suspect this level of apparent growth impairment reflects normal periods of relative stasis and not true impairment.

Recent studies suggest DMARDs and biologic agents mitigate growth impairments in children with JIA [6, 7]. It is not clear whether this is due to better control of the disease, reduced doses of corticosteroids, or both. One non-controlled trial of anakinra monotherapy in systemic arthritis avoided use of corticosteroids in 13 of 20 children [27]. The 2013 update of the American College of Rheumatology juvenile arthritis treatment recommendations includes initial monotherapy with biologics as an option in children with systemic arthritis [28]. It will be important to ascertain if early monotherapy with biologics prevents the growth impairments observed in patients with systemic JIA in our cohort.

### Study strengths and limitations

To our knowledge, this is the largest prospective study of growth in children with JIA published to date. It reflects more recent treatment practices than previous studies and we analysed growth in multiple ways to ensure our findings were robust. Nonetheless, our data should be interpreted in light of five limitations. First, although Z-scores are age- and sex-standardized this may not fully adjust for the effect of age and pubertal status on the child’s susceptibility to growth impairment, and we did not collect data on pubertal status in this cohort. Second, because procedures to measure height and weight were not standardized and the stadiometers and balances were not cross-calibrated, some differences in Z-scores may be due to measurement variability. Third, cumulative corticosteroids and cumulative disease activity in the previous 6 months were based on few measurements, since we did not use corticosteroid diaries and disease activity was only assessed at clinic visits. Fourth, we did not report on localized growth impairments such as leg length discrepancy or jaw asymmetry, which may occur in juvenile arthritis. Finally, our estimates refer to 3 years after diagnosis, and the risk of growth impairments may change with longer follow-up. It is important to emphasize that the reported growth impairments may be transitory and that final height and weight may be normal due to catch up growth and delayed puberty. Most children in this cohort had not completed their growth at the last assessment, and we had no parental height data to allow calculation of genetic growth potential.

### Implications for practice

Families of children with JIA should know that with modern treatments, their child’s disease will not substantially affect growth and weight gain, unless the child has systemic JIA, requires prolonged corticosteroid use (>3 months) and/or has uncontrolled disease. Families of children with systemic arthritis should know that systemic corticosteroids may control the disease, but at the cost of potential growth impairment; a 1 in 10 risk of short stature and a 1 in 3 risk of obesity within 3 years of diagnosis. Practitioners should be wary of prolonged corticosteroid use and our results quantify their effect on growth.

## Conclusions

Most children with JIA in this cohort grew and gained weight at a pace similar to modern North American populations. Children in the most frequent JIA categories grew well, but about 1 in 10 children who had systemic arthritis, uncontrolled disease activity, and/or required prolonged use of systemic corticosteroids were at risk of growth impairment. These children may benefit from earlier aggressive therapy to help prevent growth impairment, or early consultation with a pediatric endocrinologist to mitigate growth impairment by other means.

## Additional file

Additional file 1: Growth and Weight Gain in Juvenile Arthritis. (DOCX 2958 kb)

## Abbreviations

BMI: Body Mass Index
DMARDs: Disease modifying Antirheumatic drugs
JIA: Juvenile idiopathic arthritis
ReACCh-Out: The Research in Arthritis in Canadian Children Emphasizing Outcomes Cohort
RF: Rheumatoid factor
