# Supplementary material for: Growth and weight gain in children with juvenile idiopathic arthritis: results from the ReACCh-Out cohort
Source: Pediatr Rheumatol Online J. 2017 Aug 22;15:68. doi: 10.1186/s12969-017-0196-7 (PMC5567720; doi:10.1186/s12969-017-0196-7)
Supplement: Additional file 1: — Growth and Weight Gain in Juvenile Arthritis. (DOCX 2958 kb) [file 12969_2017_196_MOESM1_ESM.docx]

**Guzman J, Kerr T, Ward LM et al: Growth and Weight Gain in Juvenile Arthritis**

**Supplementary Material:**

1. Detail of methods

2. Supplementary Figures S1 to S4

3. Supplementary Tables S1 to S5

**Detail of methods**

At each study visit, children were examined by a pediatric rheumatologist. Most clinics were located within tertiary academic paediatric hospitals and used a wall mounted stadiometer and a digital balance to obtain height and weight measurements. The study protocol did not dictate specific techniques or tools to obtain these measurements.

Clinically inactive disease was defined as an active joint count of 0, absence of systemic manifestations in those with systemic arthritis, absence of enthesitis in those with enthesitis-related arthritis or psoriatic arthritis, absence of uveitis, and a physician global assessment of disease activity <1 cm. This definition was based on that of Wallace et al [17], with modifications [3]. An active joint was defined as swelling within a joint or painful limitation of movement [2].

Children were categorized as non-users of corticosteroids, transitory users or prolonged users. Transitory users were children who received systemic corticosteroids for 3 months or less shortly after diagnosis, the so called “corticosteroid bridge” to provide rapid disease control, as DMARDS may require 6 to 12 weeks to have an effect. Prolonged users, defined as more than 3 months of cumulative corticosteroid use during the study, included children who were prescribed a “corticosteroid bridge” but continued corticosteroids beyond 3 months, those who required re-introduction of corticosteroids at a later date and those in whom systemic corticosteroids were intended as a main treatment (usually children with systemic arthritis).

**Mixed effects models**

Mixed effects models were implemented in STATA as described for growth curve modelling by Rabe-Hesketh and Skrondal [20]. Based on examination of individual trajectory plots and the trajectories of the mean z-score for height and BMI shown in Figure 2, curvilinear models with initial rapid change that then stabilizes were chosen. This was accomplished by adding both a linear and a quadratic term for time in all models, expressed as weeks since diagnosis. As there was substantial variation across children in the starting point and the slope of individual trajectories of the z-score over time, models included random intercept and random slope.

Since the effect of corticosteroids and disease activity on growth may take months to become apparent, and height and weight measurements were obtained every 6 months for the first 2 years and yearly thereafter; we elected to model the cumulative dose of corticosteroids and cumulative disease activity in the 6 months before the height and weight measurement. Cumulative corticosteroid dose and cumulative disease activity were calculated as area under the curve using the trapezoid method; the cumulative dose between two consecutive visits *a* and *b* was calculated as the mean dose (*a+b/2*), multiplied by the number of elapsed weeks in between the visits. Then, all such intervals that occurred within 182 days before the date of measurement were added. For subjects receiving corticosteroids at enrolment (median of 0.5 months after diagnosis) it was assumed that corticosteroids were started at diagnosis at the same dose. For calculation of cumulative disease activity at enrolment, disease activity was assumed to be zero the day before the first JIA symptom, increasing linearly up to the level observed at enrolment.

To facilitate interpretation of the resulting beta coefficients for corticosteroids and disease activity, model coefficients per week were multiplied by 26 (the number of weeks in 6 months). This way, each unit change in cumulative corticosteroids shown in tables corresponds to one mg per kg of body weight of daily prednisone equivalents maintained for six months; and each unit of change in cumulative disease activity corresponds to 1 cm in the 10-cm physician global assessment of disease activity maintained for 6 months.

We were interested in measuring the impact of corticosteroids and disease activity on growth relative to each other after adjusting for other covariates likely associated with growth. To accomplish this, adjusted models included cumulative corticosteroids and cumulative disease activity in previous six months as time-variant variables, and parental education (as a proxy for socioeconomic status), primary ethnicity and JIA category as time-invariant variables.

**Sensitivity analyses**

To assess the robustness of our findings we did the following analyses: 1) Repeated calculations of the incidence of growth impairments using the Centers for Disease Control growth standards instead of the World Health Organization standards; 2) Repeated calculations including only children <7 years old at diagnosis, to ascertain impact of JIA on growth irrespective of pubertal delay, as pubertal status was not reported in this cohort; 3) Estimated the probability of growth acceleration (an increase of 1.0 height Z-scores or more relative to baseline), tall stature (a height above the 97.5^th^ percentile for age and sex), poor weight gain (a decrease of 1.0 BMI Z-scores or more relative to baseline) and thinness (a BMI below the 5^th^ percentile for age and sex) to assess the degree to which the reported incidence of growth impairments could be due to normal variability and our choice of cut-offs, rather than a true impact of the disease; 4) Repeated calculations for mixed effects models using the active joint count (the number of joints with swelling or painful limitation of movement, out of 71 joints examined) or the Juvenile Arthritis Disease Activity Score (the sum of physician assessment of disease activity, parent assessment of impact of the disease and active joint count) [21] as measures of disease activity, instead of the physician global assessment.

**Figure S1:** Confidence intervals of the mean height Z-scores for enthesitis-related and systemic arthritis built using quadratic fit equations. The thick lines represent the mean and the fine lines represent 95%CI.


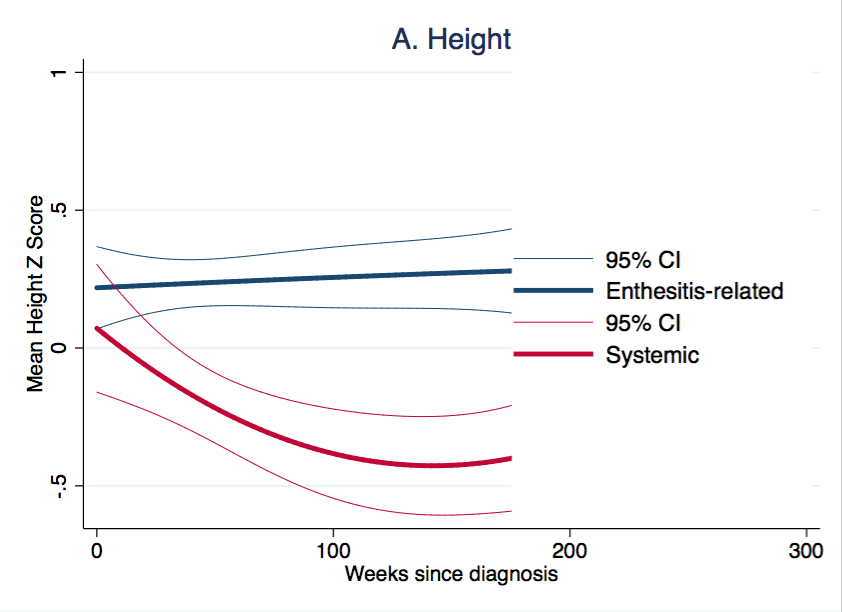


**Figure S2:** Trajectories of mean disease activity, corticosteroid dose in prednisone equivalents, height Z-scores and BMI Z-scores in each JIA category produced using locally weighted scatter plot smoothing. Disease activity was assigned by physicians in a 0 to 10 cm scale and divided by 10 to have comparable scaling to the other measures.


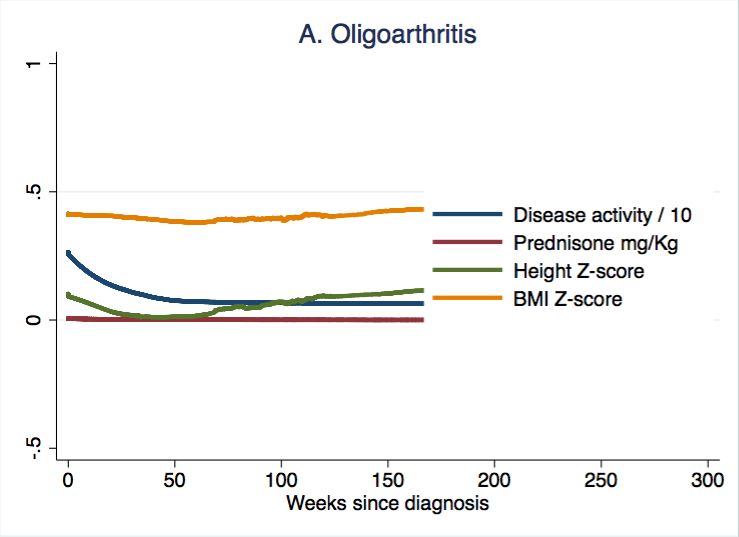

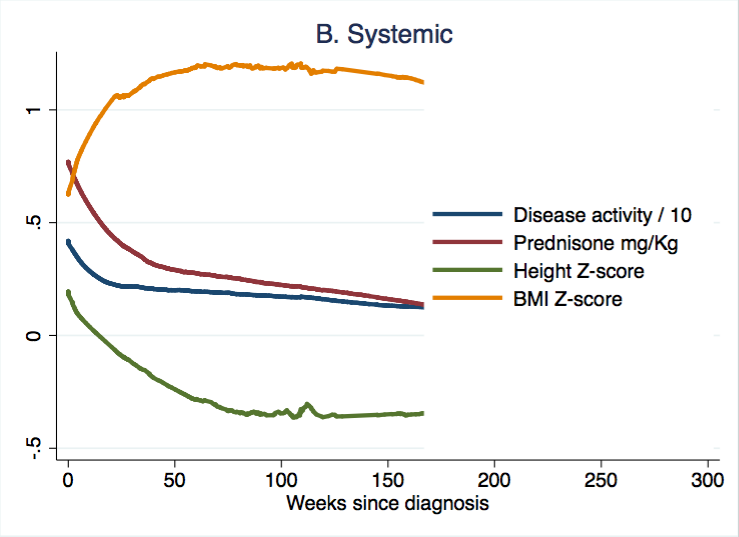

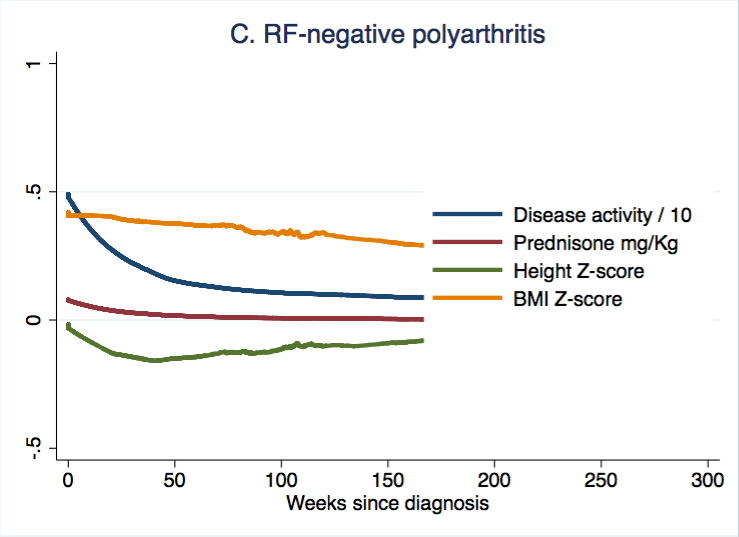

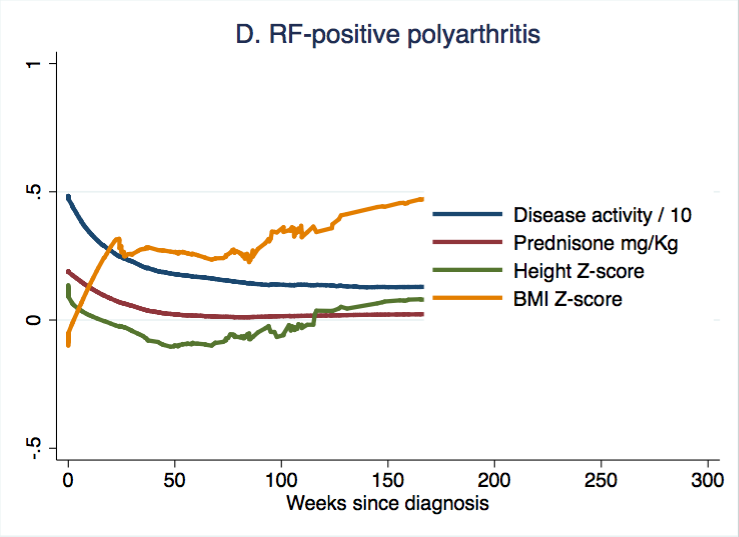

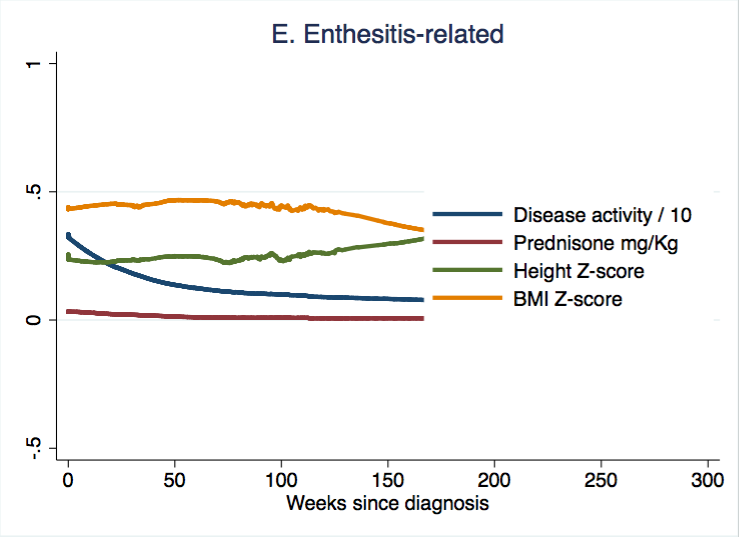

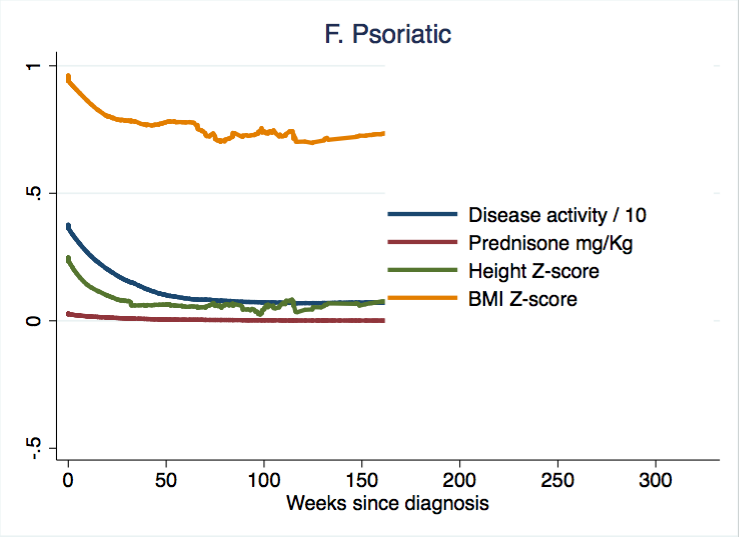


**Figure S3:** Kaplan Meier curves of the cumulative incidence of growth delay, short stature, weight gain and obesity according to corticosteroid use.

**Figure S4:** Kaplan Meier curves of the cumulative incidence of growth delay, short stature, weight gain and obesity for children who attained inactive disease within one year of diagnosis (early responders) and those who did not.

**Table S1:** Number of children providing data at each study visit by Juvenile Idiopathic Arthritis (JIA) category

| JIA Category | Visit interval (months) | | | | | | | |
| --- | --- | --- | --- | --- | --- | --- | --- | --- |
|  | 0 | 6 | 12 | 18 | 24 | 36 | 48 | 60 |
| All | 1147 | 1036 | 985 | 877 | 814 | 564 | 317 | 161 |
| Oligoarthritis | 443 | 394 | 370 | 327 | 316 | 205 | 120 | 68 |
| RF-negative polyarthritis | 232 | 215 | 205 | 186 | 166 | 130 | 77 | 40 |
| Enthesitis-related arthritis | 164 | 146 | 139 | 128 | 111 | 75 | 23 | 12 |
| Systemic arthritis | 77 | 73 | 68 | 58 | 58 | 45 | 30 | 11 |
| Psoriatic arthritis | 70 | 63 | 53 | 49 | 47 | 36 | 21 | 7 |
| RF-positive polyarthritis | 44 | 43 | 42 | 36 | 32 | 16 | 11 | 7 |
| Undifferentiated | 117 | 102 | 108 | 93 | 84 | 57 | 35 | 16 |

**Table S2:** Estimates of the cumulative incidence of growth delay, short stature, excessive weight gain and obesity within 1 and 3 years after diagnosis in seven JIA categories when **using the Centers for Disease Control growth charts.**

| JIA Category | Growth delay | | Short stature | | Excessive weight gain | | Obesity | |
| --- | --- | --- | --- | --- | --- | --- | --- | --- |
|  | Within  1 year | Within  3 years | Within  1 year | Within  3 years | Within  1 year | Within  3 years | Within  1 year | Within  3 years |
| All | 2.7  (1.9-3.9) | 8.1  (6.4-10.2) | 1.4  (0.8-2.3) | 3.1  (2.1-4.5) | 4.7  (3.6-6.1) | 10.3  (8.4-12.6) | 3.2  (2.3-4.5) | 7.5  (5.9-9.6) |
| Systemic arthritis | 8.1  (3.7-17.2) | 24.2  (15.5-36.7) | 2.9  (0.7-10.9) | 9.5  (4.3-20.2) | 15.9  (9.4-26.3) | 20.8  (13.0-32.3) | 16.0  (8.9-27.7) | 26.6  (16.8-40.5) |
| Oligoarthritis | 2.9  (1.6-5.0) | 6.1  (4.0-9.2) | 1.0  (0.4-2.5) | 2.1  (1.0-4.1) | 3.5  (2.1-5.8) | 11.7  (8.6-15.9) | 2.8  (1.6-5.0) | 6.4  (4.2-9.9) |
| RF-negative polyarthritis | 3.1  (1.5-6.4) | 6.2  (3.5-10.8) | 0.9  (0.2-3.5) | 1.4  (0.4-4.2) | 3.6  (1.8-7.0) | 7.9  (4.7-13.2) | 1.9  (0.7-5.0) | 6.7  (3.8-11.5) |
| RF-positive polyarthritis | --- | 9.9  (3.1-28.7) | --- | --- | 9.1  (3.5-22.4) | 16.1  (7.3-33.3) | 2.6  (0.4-16.8) | 2.6  (0.4-16.8) |
| Psoriatic arthritis | 1.4  (0.2-7.7) | 11.2  (4.7-25.3) | 2.9  (0.7-11.2) | 12.0  (5.4-25.4) | 3.0  (0.7-11.4) | 7.4  (2.8-19.0) | --- | 11.4  (4.9-25.3) |
| Enthesitis-related arthritis | 1.2  (0.3-4.7) | 7.2  (3.6-14.4) | 1.8  (0.6-5.6) | 1.8  (0.6-5.6) | 4.3  (2.1-8.9) | 8.1  (4.4-14.6) | 2.9  (1.1-7.5) | 5.7  (2.7-11.7) |
| Undifferentiated | 1.8  (0.5-7.1) | 7.6  (3.3-16.7) | 1.9  (0.5-7.3) | 3.6  (1.1-11.2) | 2.6  (0.9-8.0) | 5.7  (2.6-12.2) | 1.9  (0.5-7.5) | 4.1  (1.6-10.7) |

Growth delay: a decrease of 1.0 or more in height Z-score relative to the z-score of that child at enrolment. Short stature: a height below the 2.5^th^ percentile for age and sex. Excessive weight gain: an increase of 1.0 or more in BMI Z-score relative to the z-score of that child at enrolment. Obesity: a BMI above the 95^th^ percentile for age and sex.

Numbers are % and (95% CI).

--- = Cumulative incidence could not be calculated as no child had experienced the growth impairment at this time

**Table S3:** Estimates of the cumulative incidence of growth acceleration, tall stature, poor weight gain and thinness within 1 and 3 years after diagnosis in seven JIA categories. *

| JIA Category | Growth acceleration | | Tall stature | | Poor weight gain | | Thinness | |
| --- | --- | --- | --- | --- | --- | --- | --- | --- |
|  | Within  1 year | Within  3 years | Within  1 year | Within  3 years | Within  1 year | Within  3 years | Within  1 year | Within  3 years |
| All | 1.6  (1.0-2.5) | 4.5  (3.3-6.1) | 0.8  (0.4-1.6) | 2.3  (1.5-3.7) | 3.8  (2.8-5.1) | 13.9  (11.7-16.5) | 1.4  (0.8-2.3) | 4.5  (3.3-6.3) |
| Systemic arthritis | 1.3  (0.2-9.1) | 3.5  (0.8-13.7) | --- | 2.0  (0.3-13.4) | 2.7  (0.7-10.5) | 13.1  (6.6-24.8) | --- | --- |
| Oligoarthritis | 2.1  (1.1-4.0) | 3.4  (2.0-5.8) | 0.2  (0.0-1.7) | 2.5  (1.3-5.1) | 5.9  (4.0-8.6) | 14.7  (11.3-19.1) | 1.9  (1.0-3.8) | 3.4  (1.9-6.1) |
| RF-negative polyarthritis | 1.8  (0.7-4.7) | 4.1  (2.0-8.1) | 1.4  (0.4-4.2) | 1.9  (0.7-4.9) | 2.2  (0.9-5.3) | 9.9  (6.3-15.2) | 0.5  (0.1-3.2) | 5.0  (2.6-9.6) |
| RF-positive polyarthritis | --- | 6.1  (1.5-12.3) | --- | --- | 2.3  (0.3-15.4) | 11.9  (3.6-35.6) | --- | --- |
| Psoriatic arthritis | --- | --- | 1.6  (0.2-10.9) | 1.6  (0.2-10.9) | --- | 13.7  (6.8-26.7) | --- | 2.3  (0.3-15.4) |
| Enthesitis-related arthritis | 1.2  (0.3-4.6) | 7.7  (3.8-15.4) | 1.3  (0.3-5.0) | 3.2  (0.9-11.2) | 2.6  (1.0-6.7) | 14.9  (9.2-23.7) | 2.0  (0.6-6.1) | 7.8  (3.5-16.7) |
| Undifferentiated | 1.7  (0.4-6.8) | 7.7  (3.7-15.8) | 1.8  (0.5-7.2) | 2.9  (0.9-8.8) | 4.4  (1.9-10.2) | 20.5  (13.0-31.6) | 2.7  (0.9-8.2) | 10.2  (5.3-19.0) |

Grow acceleration = and increase of 1.0 height Z-scores or more relative to baseline, tall stature = a height above the 97.5 percentile for age and sex, poor weight gain = a decrease in 1.0 BMI Z-scores or more relative to baseline, thinness = a BMI below the 5 percentile for age and sex.

Numbers are % and (95% CI).

--- = Cumulative incidence could not be calculated as no child had experienced the growth impairment at this time.

**Table S4:** Estimates of the cumulative incidence (%) of growth delay, short stature, excessive weight gain and obesity within 1 and 3 years after diagnosis in seven JIA categories **for children younger than 7 years at diagnosis.**

| JIA Category | Growth delay | | Short stature | | Excessive weight gain | | Obesity | |
| --- | --- | --- | --- | --- | --- | --- | --- | --- |
|  | Within  1 year | Within  3 years | Within  1 year | Within  3 years | Within  1 year | Within  3 years | Within  1 year | Within  3 years |
| All (N=443) | 7.5  (5.3-10.6) | 14.3  (10.7-18.9) | 1.6  (0.7-3.5) | 5.1  (3.0-8.4) | 5.8  (3.9-8.6) | 11.0  (8.0-15.0) | 5.5  (3.5-8.5) | 11.6  (8.2-16.1) |
| Systemic arthritis (n=40) | 11.9  (4.6-28.7) | 33.7  (19.5-54.0) | 3.2  (0.5-20.8) | 22  (10.4-42.9) | 16.2  (7.6-32.6) | 30.2  (8.2-49.6) | 27.0  (13.9-48.5) | 43.1  (10.7-66.2) |
| Oligoarthritis (n=235) | 7.4  (4.5-11.9) | 9.8  (6.2-15.5) | 1.0  (0.2-3.8) | 2.3  (0.8-6.0) | 5.3  (3.0-9.3) | 11.2  (7.2-17.2) | 3.8  (1.8-7.9) | 9.8  (5.7-16.7) |
| RF-negative polyarthritis (n=93) | 8.1  (4.0-16.3) | 15.2  (8.5-26.3) | 1.1  (0.2-7.9) | 1.1  (0.2-7.9) | 4.6  (1.8-11.9) | 6.1  (2.7-14.1) | 4.9  (1.9-12.5) | 7.9  (3.6-16.8) |
| RF-positive polyarthritis (n=6) | --- | --- | --- | --- | --- | --- | --- | --- |
| Psoriatic arthritis (n=21) | 11.1  (2.9-37.6) | 11.1  (2.9-37.6) | 5.9  (0.8-35.0) | 5.9  (0.8-35.0) | --- | --- | --- | 10.0  (1.5-52.7) |
| Enthesitis-related arthritis (n=5) | --- | --- | --- | --- | --- | --- | --- | --- |
| Undifferentiated (n=43) | 2.6  (0.4-16.8) | 10.6  (3.4-30.3) | 2.6  (0.4-17.2) | 7.5  (1.8-28.4) | 4.9  (1.2-16.2) | 7.7  (2.5-22.0) | 2.9  (0.4-19.1) | 2.9  (0.4-19.1) |

Growth delay: a decrease of 1.0 or more in height Z-score relative to the z-score of that child at enrolment. Short stature: a height below the 2.5^th^ percentile for age and sex. Excessive weight gain: an increase of 1.0 or more in BMI Z-score relative to the z-score of that child at enrolment. Obesity: a BMI above the 95^th^ percentile for age and sex.

Numbers are % and (95% CI).

--- = Cumulative incidence could not be calculated due to the small number of children younger than 7 years old in this category.

**Table S5:** Impact of corticosteroid use and disease activity on height and BMI Z-scores, (A) when disease activity is measured with the physician global assessment of disease activity, (B) when disease activity is measured with the active joint count, and (C) when disease activity is measured with the Juvenile Arthritis Disease Activity Index (JADAS)

**(A) Using physician global assessment**

|  | ***Impact on height Z-scores*** | | | | ***Impact on BMI Z-scores*** | | | |
| --- | --- | --- | --- | --- | --- | --- | --- | --- |
| **Variable** | **Unadjusted Beta coefficient**  **(95% CI)** | ***p* value** | **Adjusted Beta coefficient ***  **(95% CI)** | ***p* value** | **Unadjusted Beta coefficient**  **(95% CI)** | ***p* value** | **Adjusted Beta coefficient ***  **(95% CI)** | ***p* value** |
| Cumulative corticosteroids in previous six months ** | -0.69  (-0.82, -0.56) | <0.01 | -0.64  (-0.77, -0.50) | <0.01 | 0.79  (0.63, 0.96) | <0.01 | 0.74  (0.56, 0.92) | <0.01 |
| Cumulative physician global in previous six months*** | -0.02  (-0.03, -0.01) | <0.01 | -0.01  (-0.02, 0.00) | 0.04 | 0.00  (-0.01, 0.01) | 0.49 | -0.01  (-0.02, 0.00) | 0.17 |

**(B) Using active joint count**

|  | ***Impact on height Z-scores*** | | | | ***Impact on BMI Z-scores*** | | | |
| --- | --- | --- | --- | --- | --- | --- | --- | --- |
| **Variable** | **Unadjusted Beta coefficient**  **(95% CI)** | ***p* value** | **Adjusted Beta coefficient ***  **(95% CI)** | ***p* value** | **Unadjusted Beta coefficient**  **(95% CI)** | ***p* value** | **Adjusted Beta coefficient ***  **(95% CI)** | ***p* value** |
| Cumulative corticosteroids in previous six months ** | -0.69  (-0.82, -0.56) | <0.01 | -0.645  (-0.78, -0.51) | <0.01 | 0.79  (0.63, 0.96) | <0.01 | 0.75  (0.57, 0.93) | <0.01 |
| Cumulative active joint count in previous six months*** | -0.01  (-0.015, -0.006) | <0.01 | -0.006  (-0.011, -0.001) | 0.02 | -0.004  (-0.010, 0.00) | 0.22 | -0.005  (-0.012, 0.00) | 0.09 |

**(C) Using the Juvenile Arthritis Disease Activity Score**

|  | ***Impact on height Z-scores*** | | | | ***Impact on BMI Z-scores*** | | | |
| --- | --- | --- | --- | --- | --- | --- | --- | --- |
| **Variable** | **Unadjusted Beta coefficient**  **(95% CI)** | ***p* value** | **Adjusted Beta coefficient ***  **(95% CI)** | ***p* value** | **Unadjusted Beta coefficient**  **(95% CI)** | ***p* value** | **Adjusted Beta coefficient ***  **(95% CI)** | ***p* value** |
| Cumulative corticosteroids in previous six months ** | -0.69  (-0.82, -0.56) | <0.01 | -0.66  (-0.83, -0.50) | <0.01 | 0.79  (0.63, 0.96) | <0.01 | 0.85  (0.63, 1.06) | <0.01 |
| Cumulative JADAS in previous six months*** | -0.003  (-0.006, 0.00) | 0.02 | -0.003  (-0.005, 0.00) | 0.07 | 0.001  (-0.002, 0.005) | 0.39 | -0.000  (-0.004, 0.003) | 0.93 |

* Unadjusted Beta coefficients were obtained from mixed effect models including only one independent variable at a time. Adjusted beta coefficients are from a mixed effects model where cumulative corticosteroids in previous 6 months, cumulative disease activity in previous 6 months, parental education, ethnicity and JIA category were entered together. Cumulative prednisone and disease activity were time-variant variables. All mixed effects models were run by subject and included random intercept, random slope and a quadratic term for time since diagnosis in weeks. They can be represented by: dependent variable = b1*constant + b2*time + b3*time square + b4* first independent variable + b5* second independent variable…

** Cumulative corticosteroids were calculated as area under the curve of daily prednisone equivalents per kilogram of body weight recorded at each clinic visit, using the trapezoid method; a one unit change corresponds to an increase of 1mg/Kg of prednisone equivalents per day sustained for 6 months. For the first study visit, corticosteroids were assumed to start at diagnosis.

*** Cumulative disease activity was calculated as area under the curve of the measure of disease activity recorded at each clinic visit, using the trapezoid method; a one unit change corresponds to an increase of 1 cm in the physician global assessment sustained for 6 months, one additional active joint sustained for 6 months, or an increase in one point in JADAS sustained for 6 months. For the first study visit, disease activity was assumed to be zero the day before disease onset.

JADAS: Juvenile Arthritis Disease Activity Score clinical version, the sum of the active joint count (to a maximum of 10 joints), the Physician global assessment of disease activity (0 to 10) and the parent global assessment of disease impact (0 to 10). Because the parent global assessment was only obtained at study visits, every 6 months for the first 2 years and yearly thereafter, the calculation of cumulative disease activity using JADAS is based on fewer measurements than calculations using the physician global or the active joint count, which were obtained at every clinic visit, not only at study visits.
